# Diagnostic value and immune infiltration characterization of YTHDF2 as a critical m6A regulator in osteoarthritic synovitis

**DOI:** 10.1186/s13018-023-03933-z

**Published:** 2023-07-27

**Authors:** Ashuai Bian, Changming Wang, Haotian Zhang, Yiqun Yan, Linlin Zhang, Wendan Cheng

**Affiliations:** 1 Department of Orthopaedics, The Second Affiliated Hospital of Anhui Medical University, Hefei, 230601 Anhui People’s Republic of China; 2 Institute of Orthopaedics, Research Center for Translational Medicine, The Second Affiliated Hospital of Anhui Medical University, Hefei, 230601 People’s Republic of China; 3 Department of Orthopedic Surgery, The First Affiliated Hospital of University of Science and Technology of China, Anhui Provincial Hospital, Hefei, 230001 Anhui People’s Republic of China

**Keywords:** Osteoarthritic synovitis, m6A regulators, Immune infiltration, Macrophage, Cluster analysis, ceRNA network

## Abstract

**Background:**

N6-methyladenosine (m6A) is a universal RNA modification pattern regulated by multiple m6A regulators. In osteoarthritis (OA), m6A regulators influence disease progression by regulating cartilage degradation. However, the function of m6A regulators in synovial tissue remains unclear. In this work, we investigated the biological significance of m6A regulators in osteoarthritic synovitis.

**Methods:**

Datasets were acquired from Gene Expression Omnibus. Differential analysis of merged data identified the differentially expressed m6A regulators. Machine learning models were used to evaluate genetic importance. To predict disease risk, a nomogram was constructed based on above m6A regulators. Cluster analysis divided the OA sample into different subgroups. Immune infiltration revealed the immune m6A regulators, which were validated using clinical samples. Eventually, a competing endogenous RNA (ceRNA) network was constructed.

**Results:**

We acquired five differentially expressed m6A regulators and a random forest model. The nomogram accurately predicted disease risk. We identified 122 differentially expressed genes between two m6A subgroups. The analysis of immune infiltration showed that YTHDF2 was an immune-related m6A regulator closely related with macrophages. In clinical samples, the protein and mRNA contents of YTHDF2 were consistent with the results of bioinformatic analysis. The ceRNA network based on YTHDF2 revealed 75 lncRNA nodes and 19 miRNA nodes.

**Conclusion:**

YTHDF2 has a high diagnostic value in the synovitis of OA and significantly influences the immune status of patients. Hence, YTHDF2, a critical m6A regulator, may provide a biomarker for diagnosis and immune therapy of osteoarthritic synovitis.

**Supplementary Information:**

The online version contains supplementary material available at 10.1186/s13018-023-03933-z.

## Introduction

Osteoarthritis (OA) is a common chronic joint disease [1]. Mechanical injury, inflammation, and immunological changes result in the destruction of all articular tissues. In addition to the cartilage degeneration, the pathological changes of OA include synovial inflammation and subchondral bone hyperplasia [2]. Osteoarthritis occurs in the synovial joint [3], resulting in chronic pain and movement disorders [4]. Joint replacement is the primary choice for end-stage OA [5]. Early intervention in OA has long been the focus of research.

RNA N6-methyladenosine (m6A) modification refers to the methylation (–CH_3_) of the sixth nitrogen atom (N) of adenine, which is the most common RNA modification pattern,. Multiple regulatory factors, called m6A regulators, are involved in m6A, leading to a dynamic and reversible pattern. The composition of m6A regulators includes m6A methyltransferase, demethylase, and m6A binding proteins. They are also called writers, erasers, and readers [6]. By regulating the generation and metabolism of RNA, m6A regulators play a part in physiological processes and disease progression [7].m6A is common in the musculoskeletal system [8, 9]. In addition, accumulating evidence shows that m6A regulators can affect the degeneration of joints [10] and inflammatory responses [11] in OA. However, previous studies neglected the synovial tissue. Synovial inflammation mediates joint damage through immune recruitment and matrix-degrading enzymes [12]. Thus, investigating the effects of m6A regulators in osteoarthritic synovitis is necessary.

The purpose of this study was to determine the gene expression of m6A regulators in synovitis of OA and evaluate their diagnostic value and association with immune factors through bioinformatic analyses. We thought this work contribute to discover new biological targets of OA.

## Methods

### Date download and processing

Microarray datasets, GSE55235, GSE55457, and GSE12021, were obtained from Gene Expression Omnibus (GEO) database (https://www.ncbi.nlm.nih.gov/geo). The relevant information about datasets is provided in Table 1. R software (version 4.2.3; https://www.r-project.org) was utilized in the bioinformatics analysis. The datasets were presented on GPL96 [HG-U133A] Affymetrix Human Genome U133A Arrays, and batch effects of merged data were removed using the SVA package (version 3.46.0) in R.

**Table 1:** GEO series information

Gene sets	Classification	Source of sample	Platform
GSE55235	10 OA VS 10 NM	Synovium of the knee joint	GPL96
GSE55457	10 OA VS 10 NM	Synovium of the knee joint	GPL96
GSE12021	10 OA VS 9 NM	Synovium of the knee joint	GPL96

OA: patients with osteoarthritis; NM: healthy control population

### Differential analysis of m6A regulatory factors

The limma package (version 3.54.2) was used to analyze the differential expression of 25 m6A regulators using the Wilcox test. The members of m6A regulators are presented in Table 2. Results of the differential analysis are presented as heat maps created using the pheatmap package (version 1.0.12). Furthermore, the cutoff criterion for OA-related m6A regulators was *P* value < 0.05, and the result was presented as boxplots using the ggpubr package (version 0.6.0).

**Table 2:** m6A regulators

Writers	WTAP, ZC3H13, RBM15, RBM15B, CBLL1, METTL3, METTL14, VIRMA
Erasers	ALKBH5, FTO
Readers	LRPPRC, HNRNPA2B1, IGFBP1, IGFBP2, IGFBP3, RBMX, ELAVL1, IGF2BP1,YTHDC1,YTHDC2,HNRNPC,FMR1,YTHDF1,YTHDF2,YTHDF3

### Machine learning models and nomogram

Random forest (RF) and support vector machines (SVM) models, based on the OA-related m6A regulators, were used for further gene selection. The accuracy of the above models was estimated through receiver operating characteristic (ROC) and residual analyses. The rms (version 6.7.0) and rmda (version 1.6) packages were utilized to construct the nomogram based on these results of machine learning model selection.

### Consistency cluster analysis

The ConsensusClusterPlus package (version 1.62.0) was applied to obtain the m6A subgroups of merged data. To quantitatively analyze the subgroups, principal component analysis (PCA) was used to verify the distribution of OA samples in subgroups and count the m6A score.

### Biological enrichment analysis between m6A subtypes

The limma package was performed for differential analysis between subtypes, and the cutoff criteria was set as |logFC|> 2 and *P*-adjust < 0.05. To investigate the biological function between m6A patterns, Gene Ontology (GO) and Kyoto Encyclopedia of Genes and Genomes (KEGG) were applied to enrichment analysis using the clusterProfiler package (version 4.6.2). The GO terms involved biological process (BP), cellular component (CC), and molecular function (MF). Only the enrichment results with *P* value < 0.05 were identified as being significant.

### Immune infiltration of m6A regulators

To analyze the extent of immune infiltration, single sample gene set enrichment analysis (ssGSEA) and CIBERSORT algorithms were used to calculate the proportions of immune cells in merged data. The correlation between m6A regulators and immune cells was estimated through Spearman analysis.

### Validation and KEGG pathways of immune-related m6A regulator

The ROC curve was performed to evaluate the diagnostic value of signature gene. We identified the gene with an area under curve (AUC) > 0.7 as a diagnostic biomarker. To investigate the biological signaling pathways of signature m6A regulator, GSEA and gene set variation analysis (GSVA) were performed to obtain the KEGG pathways with *P* value < 0.05.

### Construction of ceRNA networks

The miRanda, miRDB, and TargetScan databases were employed to predict the target microRNA (miRNA) of the signature gene. The spongScan database was used to identify the miRNA–long non-coding RNA (lncRNA) pairs. The ceRNA networks were constructed to present the relationship of messenger RNA (mRNA)–miRNA–lncRNA through Cytoscape software (version 3.10.0).

### Collection of clinical samples

Normal knee synovial tissues were obtained from donors without joint disease, and all donors underwent amputation owing to severe lower limb trauma. In addition, clinical samples of OA were procured from the synovium removed during total knee arthroplasty. The ethics committee of the Second Affiliated Hospital of Anhui Medical University approved this project. The number of project was YX2022-104. Meanwhile, all patients of this program signed informed consent.

### Western blotting (WB) and qRT-PCR experiments

Western blotting (WB) was conducted to determine the protein content of feature gene in clinical samples. Following protein sample processing, gel preparation, sample loading, electrophoresis, membrane transfer, blocking, incubation of primary and secondary antibodies, and development, the gray level analysis map of WB bands was obtained. Moreover, the mRNA content was measured via quantitative reverse transcriptase polymerase chain reaction (qRT-PCR) was used in clinical samples. The mRNA expression level was obtained through RNA extraction, RNA concentration determination, reverse transcription, preparation of primers, and construction of PCR reaction system. The primer design sequences of the characteristic genes are shown in Table 3.

**Table 3:** Primer information for characteristic genes and internal controls

Gene	Pre-primer(5′–3′)	Post primer (5′–3′)	Molecular weight(kDa)
GAPDH	GCAAAGTGGAGATTGTTGCC	TGGAAGATGGTGATGGGCTT	37
YTHDF2	ATAGGAAAAGCCAATGGAGGG	CCAAAAGGTCAAGGAAACAAAG	62

## Results

### Differentially expressed m6A regulators

**Fig. 1:**
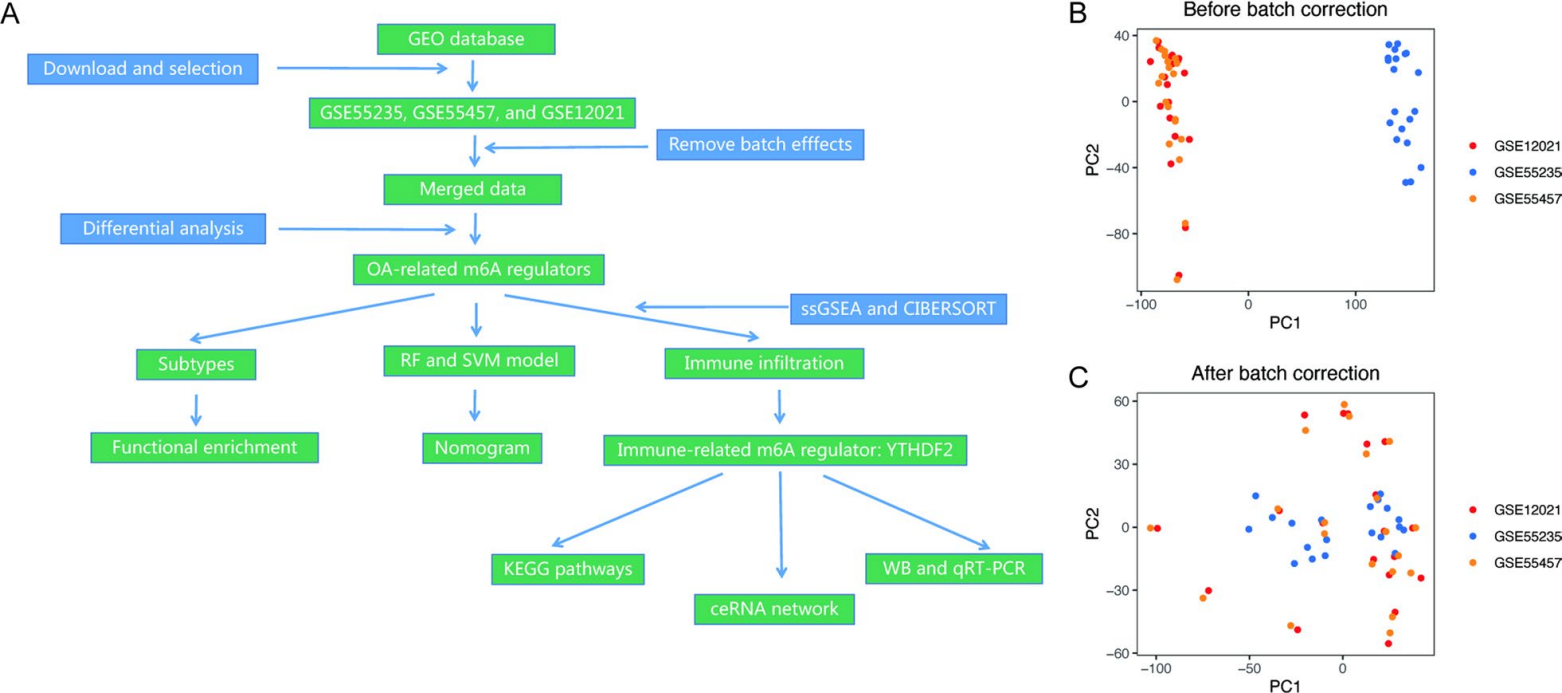
Flow chart and data preprocessing. **A** Flow chart of this study. **B** Principal component analysis (PCA) before batch correction. **C** PCA after batch correction

**Fig. 2:**
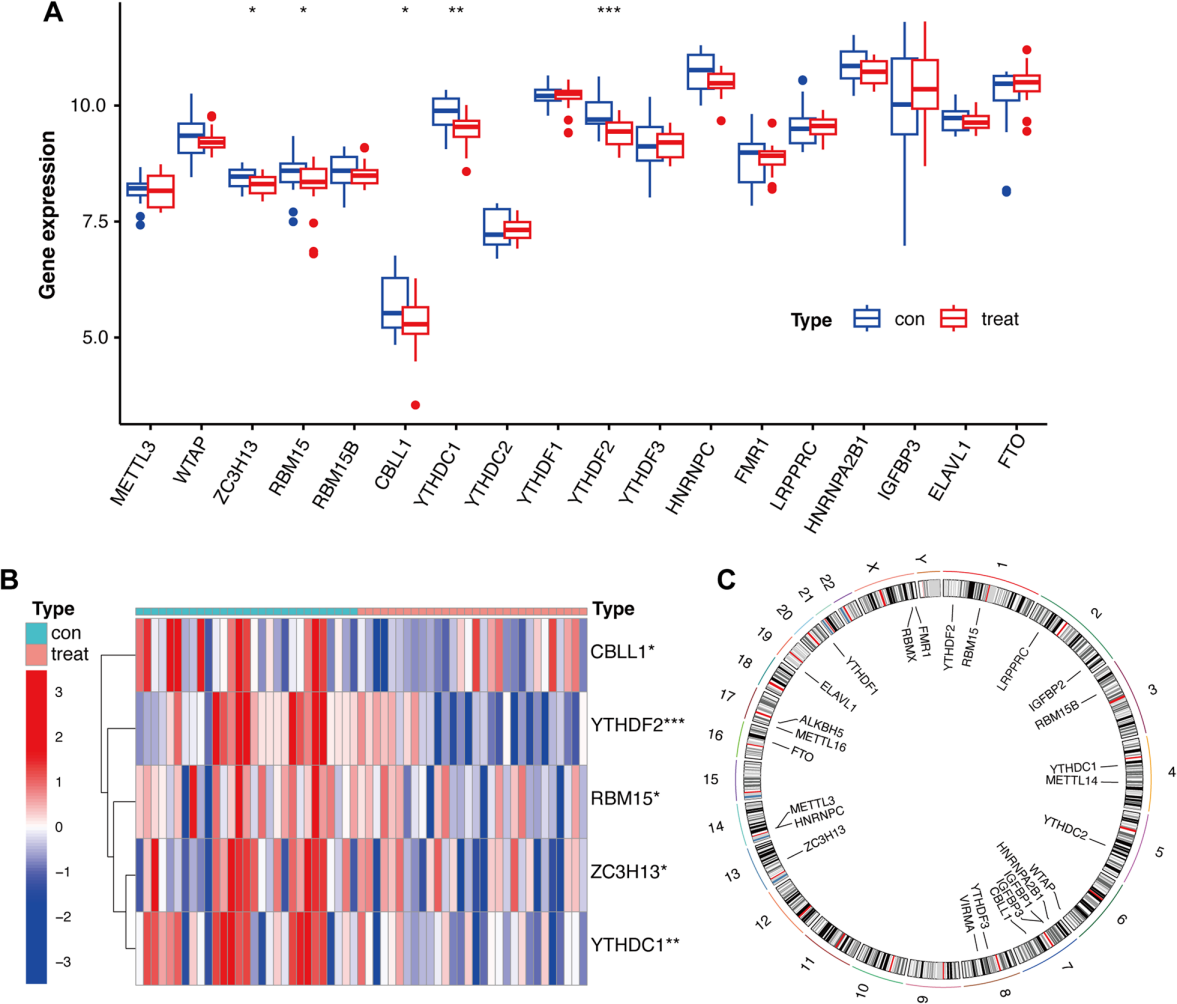
Expression differences in m6A regulators. **A** Boxplot of differences in m6A regulators between OA and control, *P < 0.05, **P < 0.01, ***P < 0.001. **B** Heat map of OA-related m6A regulators. **C** Chromosome circle diagram of 25 m6A regulators

The flow chart of data analyses is shown (Fig. 1A). After batch correction of the datasets from GSE55235, GSE55457, and GSE12021 (Fig. 1B, C), differentially expressed m6A regulators were identified (Fig. 2A). Figure 2B shows the heatmap of m6A regulator expression. Five differentially expressed m6A regulators included three writers (CBLL1, RBM15, and ZC3H13) and two readers (YTHDF2 and YTHDC1). Moreover, these OA-related m6A regulators were all downregulated.

### Model selection and construction of nomogram

**Fig. 3:**
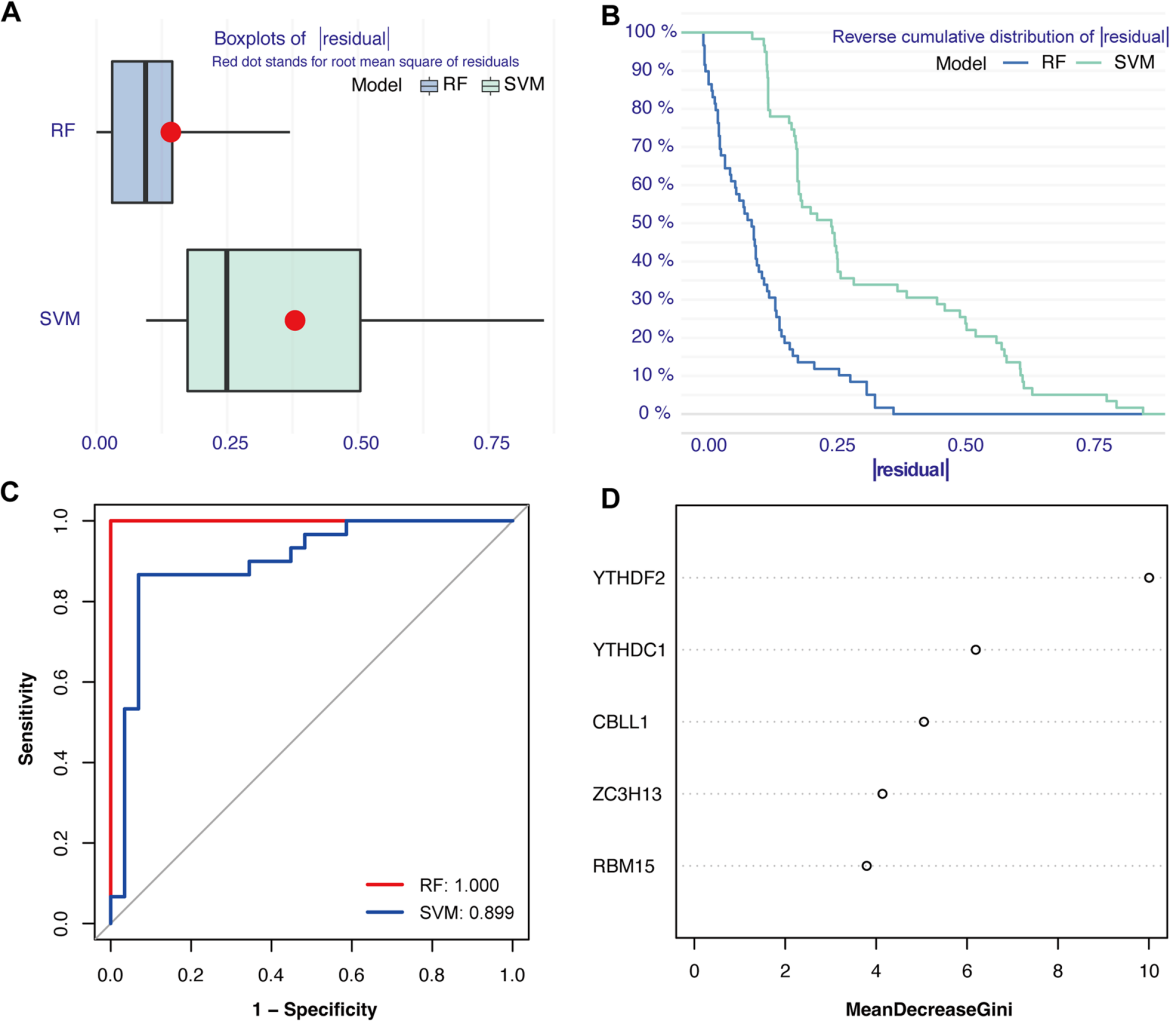
Construction of random forest (RF) and support vector machine (SVM). **A** Residual boxplots of RF and SVM. **B** Residual reverse cumulative distribution of RF and SVM. **C** Receiver operating characteristic curves of RF and SVM. **D** Importance score of m6A regulators in the accordance with RF

**Fig. 4:**
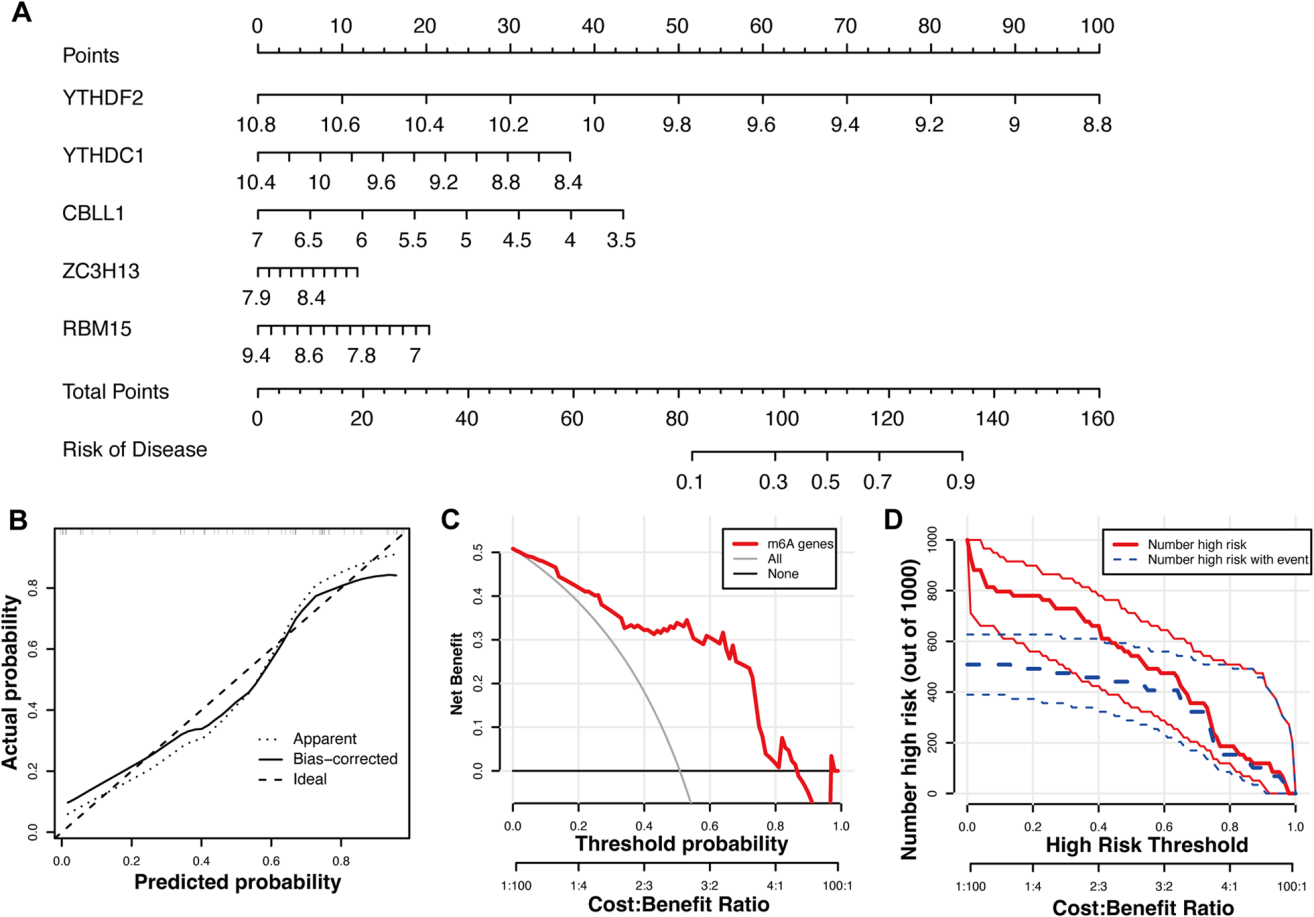
Establishment of nomogram. **A** Nomogram of five osteoarthritis-related m6A regulators. **B** Calibration curve evaluates the prediction accuracy of nomogram. **C** Decision curve assesses the predictive ability of nomogram. **D** Clinical impact curve evaluates the clinical value of nomogram

As shown in the boxplots of residuals (Fig. 3A) along with reverse cumulative distribution of residual (Fig. 3B), the residuals of RF were less than those of SVM. In addition, ROC curves (Fig. 3C) showed that the accuracy of the RF model was higher than that of SVM. Thus, the RF model was more suitable for evaluating the importance score of OA-related m6A regulators. We acquired five genes with score > 2 to construct the nomogram (Fig. 4A). Subsequently, calibration (Fig. 4B) and decision curves (Fig. 4C) showed that the prediction accuracy of the nomogram was high, and clinical impact curves verified its clinical value (Fig. 4D).

### Classification of osteoarthritic synovitis

**Fig. 5:**
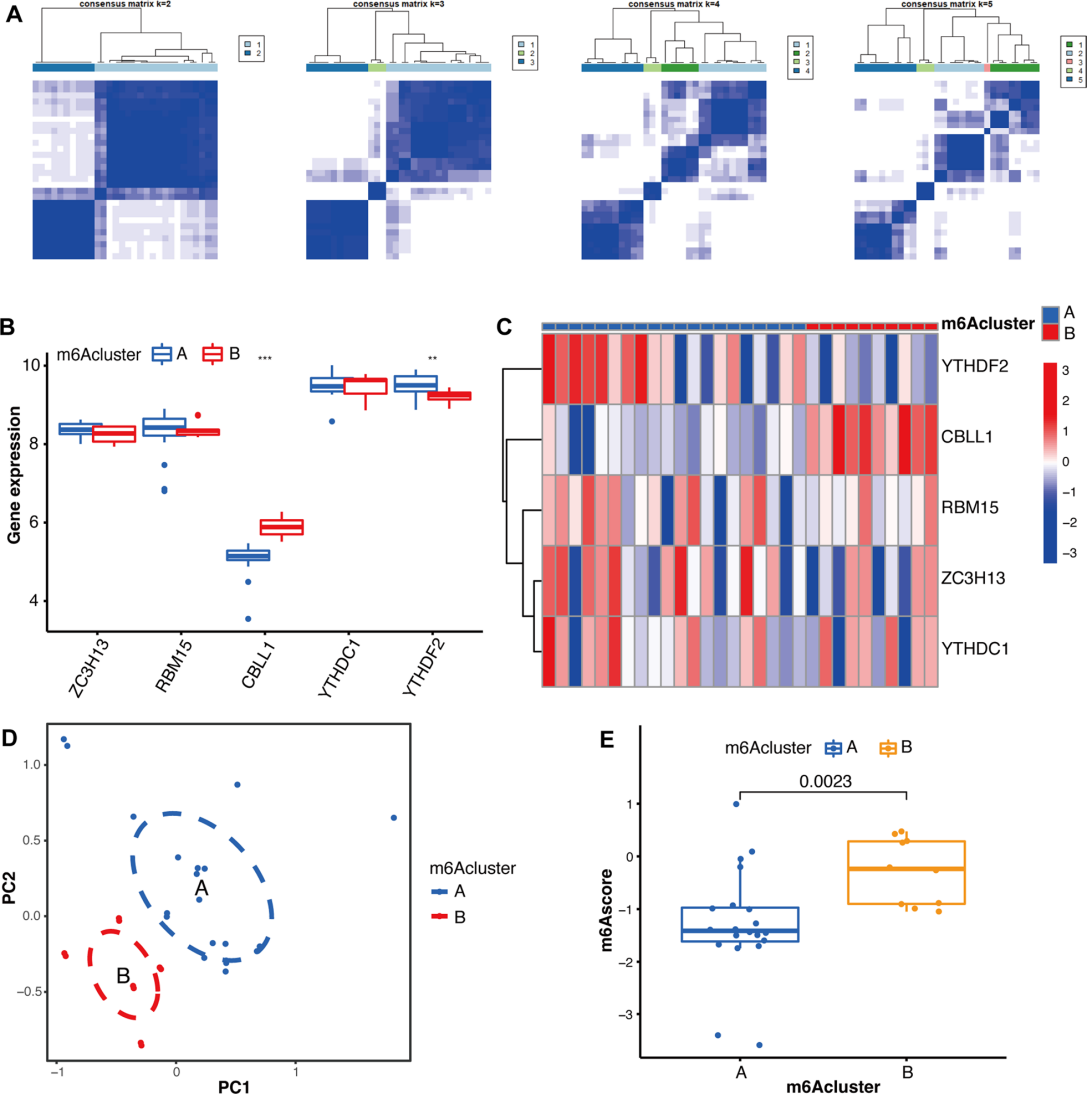
Cluster analysis of osteoarthritis (OA) samples based on m6A regulators. **A** Consistency matrix plot for *K* = 2–5. **B** Boxplot of differential expression of OA-related m6A regulators. **C** Heat map of differential expression of OA-related m6A regulators. **D** PCA of cluster A and cluster B. **E** Boxplot of the m6A score of the two subtypes

To investigate the cluster mediated by m6A regulators in OA, two distinct subtypes were obtained via unsupervised consensus clustering analysis. The *K* value, used to assess the optimal number of subtypes, showed that the result of cluster analysis was highly stable at *K* = 2 (Fig. 5A). Boxplots and heat maps (Fig. 5B, C) revealed the differential analysis of five OA-related m6A regulators among both m6A subtypes. Furthermore, PCA verified the rationality of our classification, and the differences between both subtypes were quantified through m6A score (Fig. 5D, E).

**Fig. 6:**
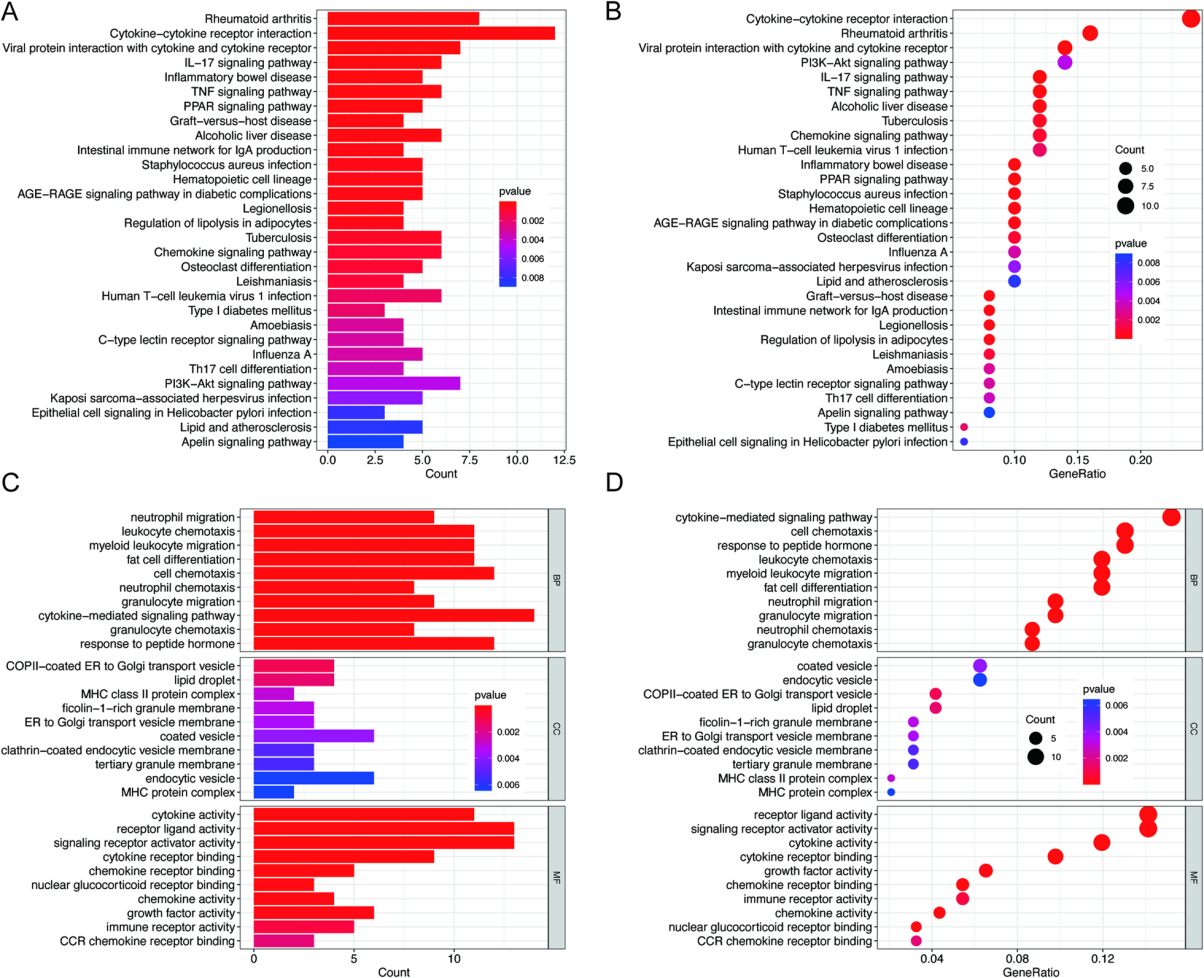
Gene Ontology (GO) and Kyoto Encyclopedia of Genes and Genomes (KEGG) between m6A clusters. **A** Bar plot of KEGG. **B** Bubble plot of KEGG. **C** Bar plot of GO. **D** Bubble plot of GO

Between the two subtypes, namely cluster A and B, we obtained 122 differentially expressed genes, and the list of differential genes is provided in Additional file 1. Based on these genes, biological enrichment analysis was performed through GO and KEGG. The KEGG analysis results showed that biological pathways included cytokine–cytokine receptor interaction, rheumatoid arthritis, and PI3K-Akt signaling pathway (Fig. 6A,B). Moreover, the results of GO contained cytokine-mediated signaling pathway, coated vesicle, receptor ligand activity, and so on (Fig. 6C, D).

### Immune infiltration of m6A regulators

**Fig. 7:**
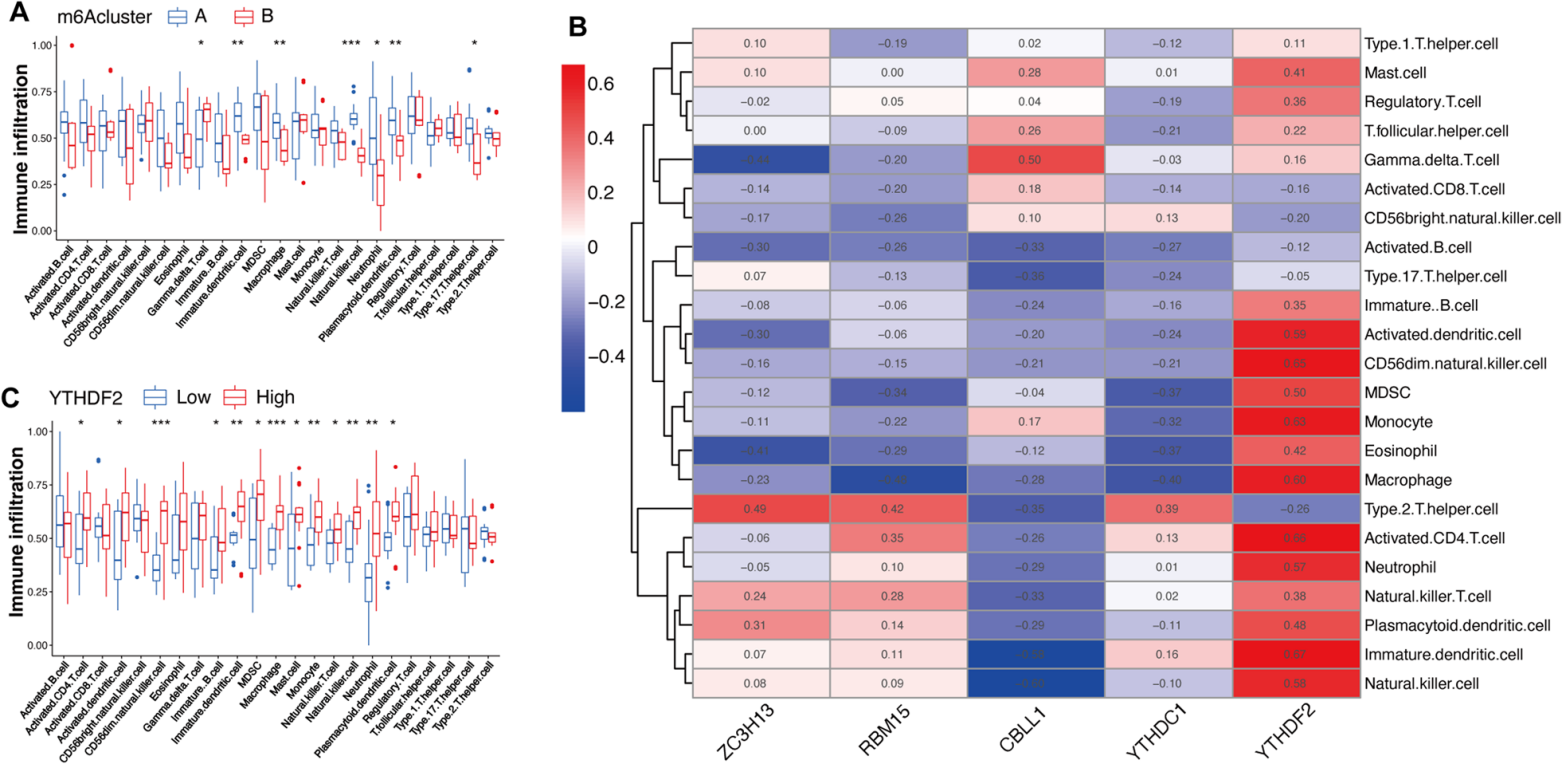
Immune infiltration of m6A regulators in osteoarthritis (OA) by ssGSEA. **A** Correlation of immune cells and m6A subtypes. **B** Association analysis of immune infiltration and five OA-related m6A regulators. **C** Correlation of immune cells and YTHDF2. *p < 0.05, **p < 0.01, and ***p < 0.001

To investigate the landscape of immune infiltration in both subgroups, the immune infiltration of 23 types of immune cells was determined through ssGSEA. The results showed differences between m6A subtypes in terms of multiple immune cells, mainly immature dendritic cells, macrophages, natural killer cells, and plasmacytoid dendritic cells (Fig. 7A). We also investigated the association between m6A regulators and immune cells, among which YTHDF2 was most associated to immune infiltration (Fig. 7B). Subsequently, according to the expression of YTHDF2, OA samples were classified into low- and high-expression groups to compare the differential expression of immune cells. The outcomes showed that there were differences in the infiltration of CD56dim natural killer cells, immature dendritic cells, macrophages, monocytes, natural killer cells, and neutrophils (Fig. 7C).

**Fig. 8:**
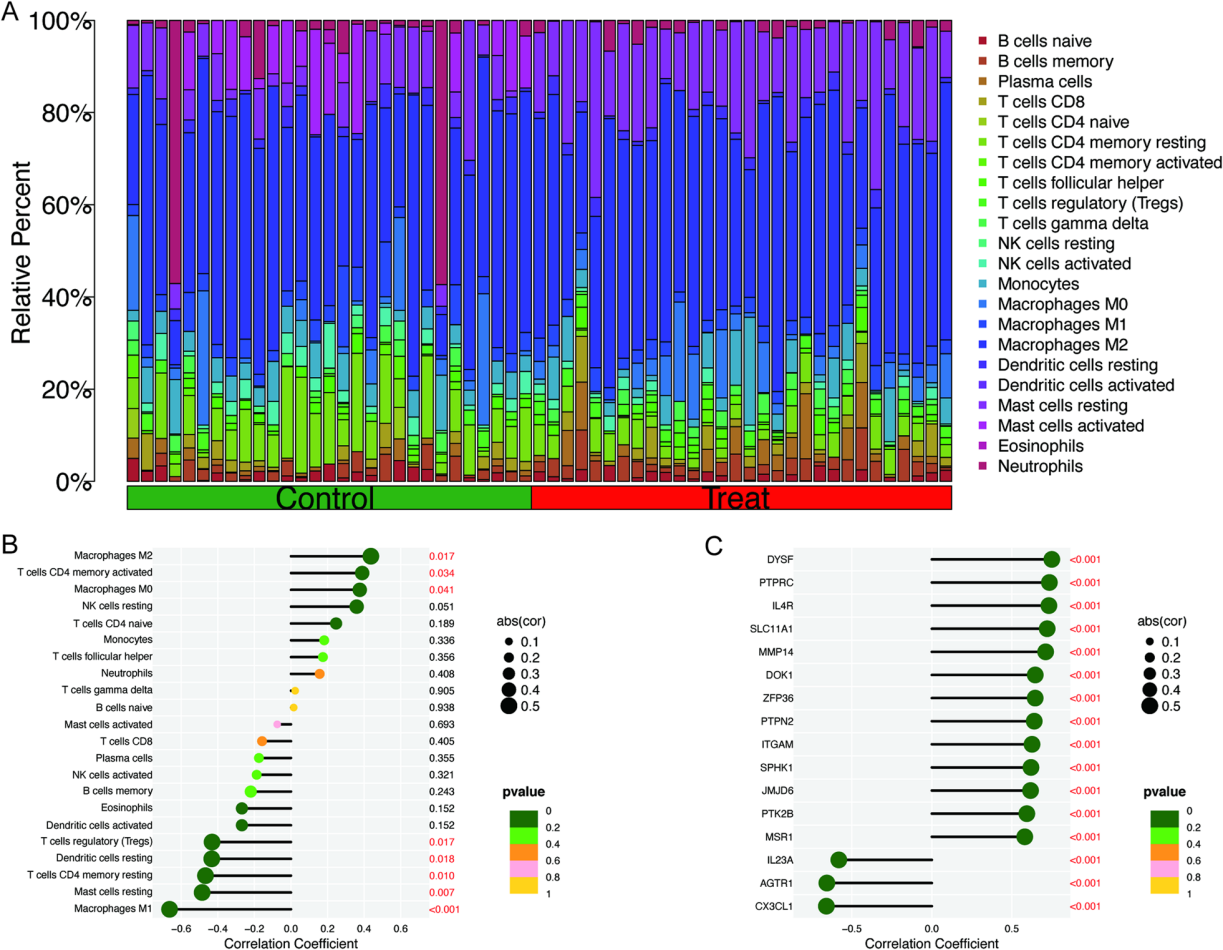
Immune infiltration of YTHDF2 in osteoarthritis (OA) by CIEERSORT. **A** Bar plot of immune cell infiltration in merged data. **B** Lollipop of correlation between YTHDF2 and immune cells. **C** Lollipop correlation between YTHDF2 and macrophage genes. Red font indicates *P* < 0.05

For further analysis of immune cell infiltration, we analyzed the infiltration of 22 types of immune cells through the CIBERSORT algorithm, among which macrophages and mast cells were predominant (Fig. 8A). Meanwhile, we analyzed the association between YTHDF2 and immune cells and found that YTHDF2 was positively correlated with M2 macrophages and negatively correlated with M1 macrophages (Fig. 8B). To investigate the association between YTHDF2 and macrophages, we obtained a list of 240 macrophage genes from the MSigDB database (http://www.gseamsigdb.org/gsea/msigdb). The list was presented in Additional file 2. A total of 16 genes were screened through Spearman analysis (Fig. 8C).

### Bological pathways of YTHDF2

**Fig. 9:**
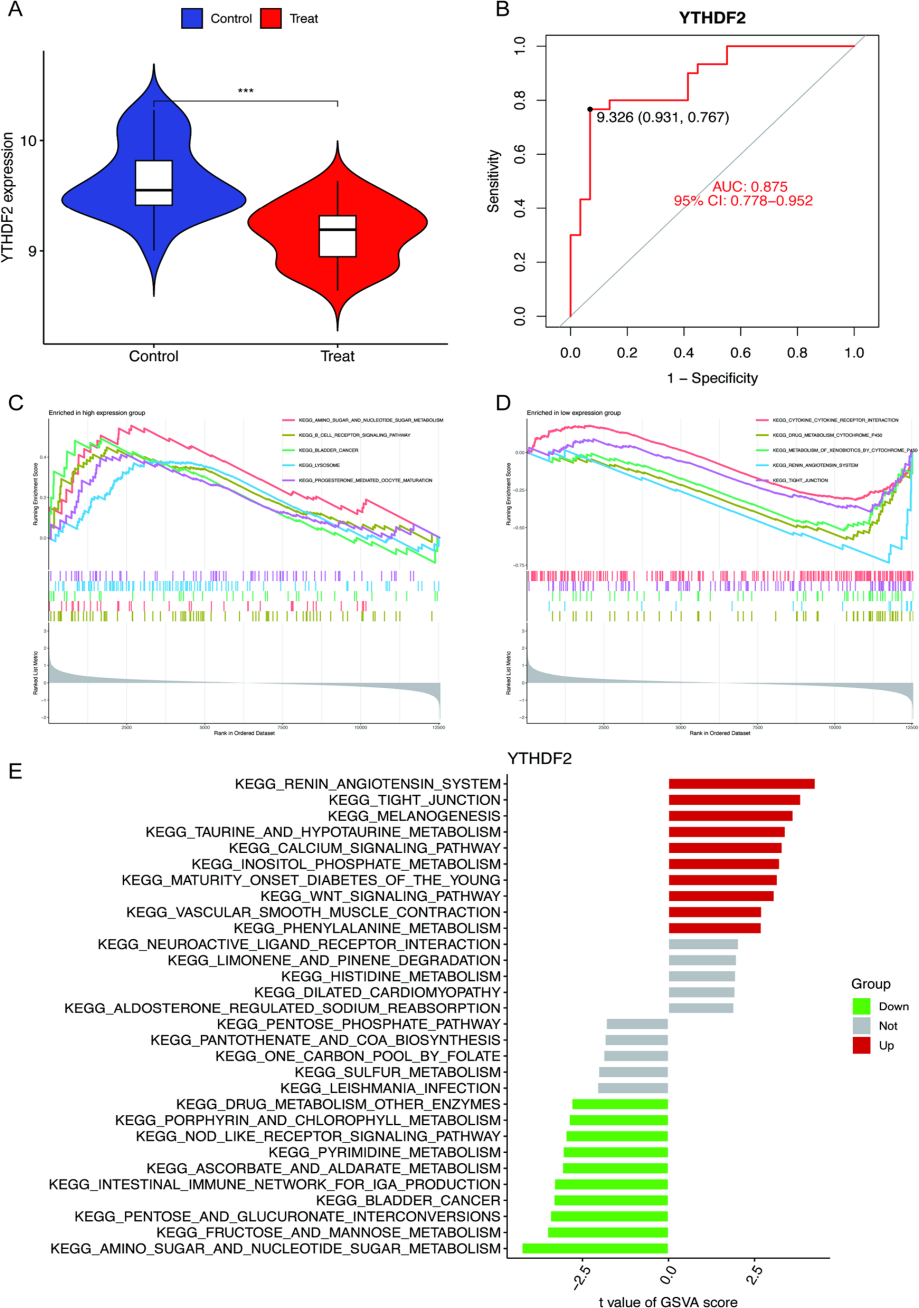
Bioinformatic validation and KEGG pathways of YTHDF2. **A** Violin plot of YTHDF2. **B** ROC of YTHDF2. **C** High expression of YTHDF2 in GSEA. **D** Low expression of YTHDF2 in GSEA. **E** KEGG pathways of YTHDF2 according to GSVA

According to the above research, YTHDF2 was the signature m6A regulator in the synovial tissue of OA. A violin plot (Fig. 9A) showed that YTHDF2 has low expression and the AUC indicated that YTHDF2 has significant diagnostic value (Fig. 9B). To research the mechanism of YTHDF2 in osteoarthritic synovitis, GSEA (Fig. 9C, D) and GSVA (Fig. 9E) were used to determine the enriched KEGG pathways. We eventually acquired some common pathways (progesterone-mediated oocyte maturation, bladder cancer, renin angiotensin system, and tight junction).

### Validation of YTHDF2 in clinical samples

**Fig. 10:**
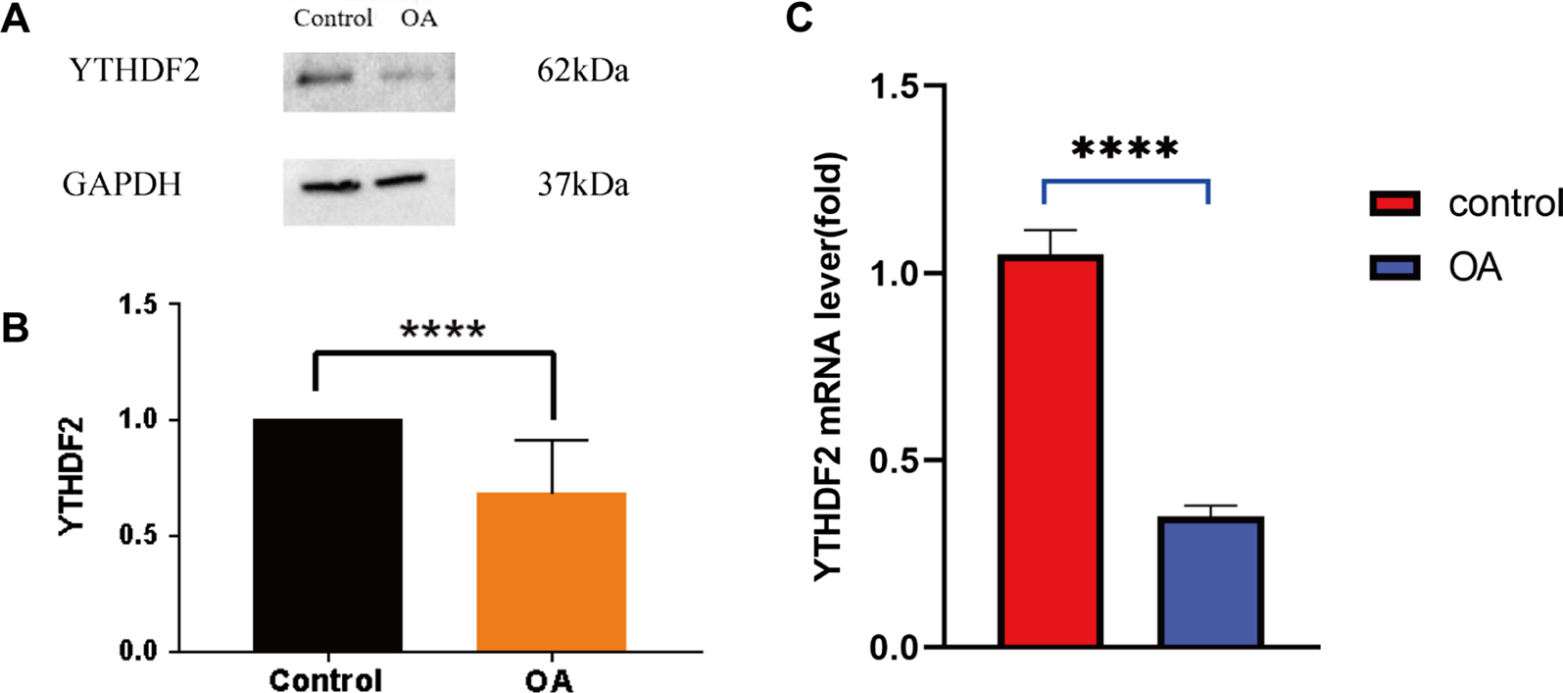
Validation of the signature m6A regulators in clinical samples. **A** Protein content of YTHDF2 in osteoarthritis (OA) and normal person by western blotting. **B** Expression of YTHDF2 protein in OA and control groups; **C** Expression level of YTHDF2 mRNA in OA and control groups. ****p < 0.0001

To verify the diagnostic value of YTHDF2, we detected its gene expression in clinical specimens via WB and qRT-PCR. Western blot analysis revealed that protein expression was significantly lower in patients with OA (Fig. 10A, B). In addition, compared with that of the control group, the mRNA content of YTHDF2 was reduced in patients with OA (Fig. 10C). The results in clinical samples were consistent with those from the bioinformatics analysis.

### Construction of the ceRNA network

**Fig. 11:**
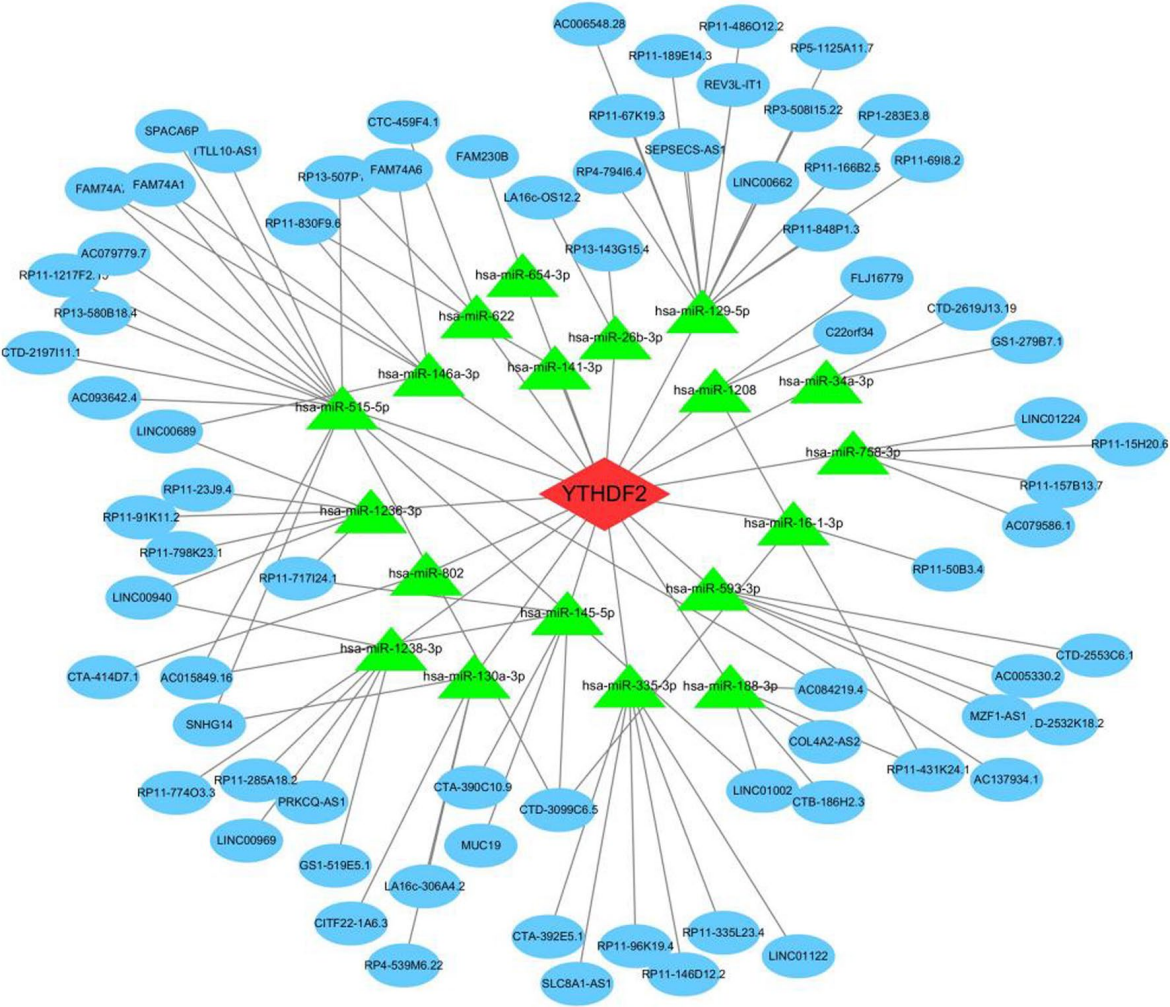
Network of ceRNA of YTHDF2. The red diamond label means mRNA, the green triangle label meas miRNA, and the blue ellipse label means lncRNA

The ceRNA network was constructed to explore the functions of ncRNAs of YTHDF2 in synovitis of OA, namely lncRNA and miRNA. This ceRNA network contained 75 lncRNA nodes, 19 miRNA nodes, 1 hub gene node, and 108 edges (Fig. 11). Details are presented in Additional file 3. Furthermore, we found two critical miRNA nodes (hsa-miR-129-5p and hsa-miR-515-5p), which had 14 and 15 targeted lncRNAs, respectively.

## Discussion

Osteoarthritis is a complex joint disease, the pathogenesis of which includes mechanical, inflammatory, immune infiltration, and metabolic factors. Thus, OA is not a degenerative disease as previously described [13]. Previous research indicates that low-grade inflammation was crucial to the pathological processes of OA [14]. In OA, synovitis is low-grade inflammation and can be observed at both early and late stages of disease [15]. Similar to rheumatoid arthritis, there are abundant immune cells in the inflammatory synovium of OA [16]. Under the influence of immune factors, degradation products stimulate synovial tissues to secrete various proinflammatory substances [17, 18]. In addition, the immune recruitment of synovitis significantly increases the risk of OA [19, 20]. Some in vivo studies have supported this finding [21, 22]. m6A is a widespread modification of RNA, regulated by m6A regulators. The network of m6A regulators includes writers, erasers, and readers [6]. By affecting the generation and metabolism of RNA, m6A regulators participate in the progression of multiple diseases. Recently, increasing evidence has shown that m6A regulators could influence the immune regulation of chondrocytes and extracellular matrix [10, 11]. Hence, research on m6A regulators in synovitis contributes to furthering our understanding of immunity in OA.

In this research, we systematically explored the diagnostic value of m6A regulators in osteoarthritic synovitis and evaluated its immune infiltration. Five significantly differentially expressed m6A regulators were screened, namely CBLL1, YTHDF2, RBM15, ZC3H13, and YTHDC1. RBM15, ZC3H13, and CBLL1 are writers responsible for generating methylation [23]. In addition, YTHDF2 and YTHDC1 both include the YTH domain, which recognizes m6A sites and regulates its metabolism as m6A readers [24]. Based on these regulators, we established a nomogram model. The results revealed that the genic expression predicts the risk of OA, especially YTHDF2. The ROC curve and validation in clinical samples also demonstrated its accuracy. By accelerating the degradation of RNA [25, 26], YTHDF2 plays an important role in disease progression. Recently, YTHDF2 was found to be able to regulate some inflammation [27]. Subsequently, cluster analysis was used to divide the OA group into two m6A subtypes. The results of enrichment between subtypes mainly included inflammation-related pathways and immune cells. Thus, we analyzed the immune cell infiltration in both m6A subgroups. The main differences were observed in immature dendritic cells, macrophages, natural killer cells, and plasmacytoid dendritic cells. Thus, innate immunity participates in the regulation of m6A subgroups of OA. Previous research has shown that innate immunity is crucial to the pathology of OA [28].

Osteoarthritis is an inflammatory disease. Low-grade inflammation has strong connection with disease development [29]. Moreover, the pathogenic mechanisms of OA include immune factors [30]. The low-grade inflammatory response is closely related to the infiltration characteristics of the immune microenvironment [31]. Recently, research has shown that m6A regulators contribute to immune response [32]. To investigate the mechanism of immunity along with m6A regulators in the synovial tissue of OA, we analyzed the correlation between m6A regulators and immune cells. The results showed that YTHDF2 is an immune-related m6A regulator. Subsequently, through the CIBERSORT algorithm, we found that YTHDF2 was positively correlated with M2 macrophages and negatively correlated with M1 macrophages. There are two classical phenotypes in synovial macrophages, namely M1 and M2. M1 macrophages are responsible for promoting inflammation, whereas the function of M2 macrophages is to antagonize inflammation [33]. Based on the downregulated YTHDF2 in OA synovitis, as shown by the bioinformatic analysis, YTHDF2 may regulate the polarization of macrophages in the synovial tissue of OA. The low gene expression of YTHDF2 resulted in attenuated anti-inflammatory and enhanced pro-inflammatory differentiation of macrophages. Previous research supports this conclusion. In the macrophage polarization model, researchers found that the classical molecular markers of M1 were upregulated after YTHDF2 knockdown. Correspondingly, those of M2 declined [34, 35]. Therefore, to investigate the specific relationship between YTHDF2 and macrophages, we obtained 240 macrophage genes from the MSigDB database and screened 16 closely related gene. These genes maybe provide new research directions for immune therapy of OA.m6A RNA modification refers to the methylation of mRNA and ncRNA [36]. Unlike mRNA, ncRNA does not have protein-coding ability. However, ncRNA also contributes to the process of gene expression [37] and participates in the pathological mechanism of many diseases [38]. In OA, some ncRNAs were found to regulate disease progression [39, 40]. miRNA and lncRNA are types of ncRNA. Based on the ceRNA theory, miRNA could combine the miRNA response element (MRE) of mRNA to regulate its degradation. In addition, some lncRNAs have the same MRE to competitively bind miRNA. Thus, lncRNAs could indirectly regulate the expression of mRNA [41]. To explore the role of the ncRNA of YTHDF2, we constructed a ceRNA network including 75 lncRNA nodes, 19 miRNA nodes, and 108 edges. We also found that hsa-miR-129-5p and hsa-miR-515-5p have the most target lncRNAs.

This study included the following limitations: First of all, the sample size needs to be enriched in future research. Second, the results were obtained from bioinformatic analysis. Thus, the lack of experiments in vivo and in vitro needs to be improved.

## Conclusion

We obtained five significant m6A regulators and established a nomogram model. Moreover, the m6A reader YTHDF2 declined in OA and was identified as potential biomarker. The validation by WB and qRT-PCR confirmed this finding. Through the evaluation of immune infiltration, we found that YTHDF2 is an immune-related m6A regulator. Further association analysis showed that YTHDF2 was negatively correlated with pro-inflammatory differentiation of macrophages. In the end, we created a ceRNA network to predict the target ncRNA of YTHDF2. These findings suggest that YTHDF2 could serve as a biomarker of osteoarthritic synovitis and may be a choice for immune therapy in future research.

## Supplementary Information

**Additional file 1:** The list of differential genes between subtypes.

**Additional file 2:** The list of macrophage-related genes.

**Additional file 3:** The ceRNA network of YTHDF2.

## Abbreviations

OA: Osteoarthritis
RF: Random forest
SVM: Support vector machine
AUC: Area under curve
ROC: Receiver operating characteristic
PCA: Principal component analysis
GO: Gene Ontology
KEGG: Kyoto Encyclopedia of Genes and Genomes
ssGSEA: Single sample gene set enrichment analysis
GSVA: Gene set variation analysis
WB: Western blotting
qRT-PCR: Quantitative reverse transcriptase polymerase chain reaction
BP: Biological process
CC: Cellular component
MF: Molecular function
miRNA: MicroRNA
lncRNA: Long noncoding RNA
mRNA: Messenger RNA
ceRNA: Competing endogenous RNA
